# The effect of laser pan-retinal photocoagulation with or without intravitreal bevacizumab injections on the OCT-measured macular choroidal thickness of eyes with proliferative diabetic retinopathy

**DOI:** 10.6061/clinics/2017(02)03

**Published:** 2017-02

**Authors:** Rony C Preti, Anibal Mutti, Daniel A Ferraz, Leandro C Zacharias, Yoshitaka Nakashima, Walter Y Takahashi, Mario L R Monteiro

**Affiliations:** Hospital das Clínicas da Faculdade de Medicina da Universidade de São Paulo, Oftalmologia, São Paulo/SP, Brazil

**Keywords:** Choroid, Choroidal Thickness, Diabetes Mellitus, Diabetic Retinopathy, Proliferative Diabetic Retinopathy, Bevacizumab, Laser Pan-Retinal Photocoagulation, Macular Edema, Optical Coherence Tomography

## Abstract

**OBJECTIVES:**

To investigate the effect of laser pan-retinal photocoagulation with or without intravitreal bevacizumab injections on macular choroidal thickness parameters in eyes with high-risk proliferative diabetic retinopathy.

**METHODS:**

High-risk proliferative diabetic retinopathy patients undergoing laser treatment were prospectively enrolled in this study. One eye was randomly selected for laser treatment combined with bevacizumab injections, study group, whereas the corresponding eye was subjected to laser treatment alone, control group. Spectral-domain optical coherence tomography with enhanced depth imaging was used to measure the macular choroidal thickness prior to and 1 month after treatment. Measurements in both groups were compared. Clinicaltrials.gov: NCT01389505.

**RESULTS:**

Nineteen patients (38 eyes) with a mean±standard deviation age of 53.4±9.3 years were evaluated, and choroidal thickness measurements for 15 patients were used for comparison. The greatest measurement before treatment was the subfoveal choroidal thickness (341.68±67.66 μm and 345.79±83.66 μm for the study and control groups, respectively). No significant difference between groups was found in terms of macular choroidal thickness measurements at baseline or after treatment. However, within-group comparisons revealed a significant increase in choroidal thickness parameters in 10 measurements in the study group and in only 5 temporal measurements in the control group when 1-month follow-up measurements were compared to baseline values.

**CONCLUSIONS:**

The macular choroidal thickness does not appear to be significantly influenced by laser treatment alone but increases significantly when associated with bevacizumab injections in patients with proliferative diabetic retinopathy and macular edema. Because bevacizumab injections reduce short-term laser pan-retinal photocoagulation-induced macular edema, our findings suggest that the choroid participates in its pathogenesis.

## INTRODUCTION

Diabetes mellitus (DM) is an important metabolic disease that is often associated with injury to several ocular structures. Diabetic retinopathy (DR) is one of its most common chronic microvascular complications 1 and one of the leading causes of new-onset blindness in industrialized and middle-income countries 2. Diabetic macular edema (DME) and proliferative diabetic retinopathy (PDR) are the primary disorders responsible for vision loss 3.

Laser pan-retinal photocoagulation (PRP) is the primary treatment modality for preventing vision loss in patients with PDR 4,5. Although PRP effectively reduces retinal neovascularization, it is associated with the release of cytokines, including vascular endothelial growth factor (VEGF), which may be related to adverse effects in the retina, including ME 6, thereby causing transient or permanent visual acuity 7-9 and contrast sensitivity loss 10. Therefore, many researchers have used anti-VEGF drugs such as intravitreal bevacizumab (IVB) injections with promising results to prevent the development of PRP-related ME 8,9.

Previous studies have indicated that DM may also affect the choroid via obstruction of the choriocapillaris, vascular degeneration, and the development of choroidal aneurysms and neovascularization that may be present even before the onset of DR 11-13. Moreover, because the outer retinal layers are essentially nourished by the choroid, some authors believe that choroidal changes play an important role in the development of DR 11,14,15.

Most previous studies evaluating possible involvement of the choroid in patients with DM were performed using indocyanine green angiography 14,16. More recently, however, optical coherence tomography (OCT) has been introduced as an important and accurate method for estimating macular CT 17.

In relation to the changes in macular CT for PDR patients treated with PRP with or without anti-VEGF, the published data are still inconsistent likely due to differences in samples and the presence or absence of DME. The only finding that has been confirmed is that PRP causes macular CT thinning in long-term follow-ups 18-22.

Despite the vast literature addressing the effects of PRP on the retina, previous OCT studies have not addressed the possible effects of PRP with or without IVB injections on the macular CT measurements of high-risk PDR patients. Thus, we prospectively investigated the effects of PRP alone or with the adjuvant use of IVB injections on macular CT measurements in patients with high-risk PDR.

## METHODS

This trial applied an interventional, prospective, masked, and randomized study design. The study followed the principles of the Declaration of Helsinki and was approved by the Institutional Review Board of the Ethics Committee of our institution. All participants signed an informed consent form. Clinicaltrials.gov: NCT01389505.

The inclusion criteria for the study were best corrected visual acuity (BCVA) ≥20/200, type 2 DM, and similar high-risk PDR in both eyes with or without DME. The exclusion criteria were pretreatment of diabetic retinopathy (laser photocoagulation or intraocular surgery), vitreous hemorrhage, vitreous-retinal interface alteration (epiretinal membrane, macular hole, or vitreoretinal traction syndrome), active external eye disease, systolic or diastolic blood pressures greater than 180 or 110 mm Hg, respectively, A1C levels exceeding 15%, and chronic renal failure.

Twenty-two patients (13 males) were enrolled in the study. All patients received a complete clinical evaluation and the following ophthalmic examinations: BCVA assessment, intraocular pressure measurements, slit-lamp examinations, fundoscopy and time-domain (*Stratus-OCT^®^*, Carl Zeiss Ophthalmic System, Inc.; Dublin, California, USA) and spectral-domain (*Spectralis^®^,* Heidelberg Engineering, Heidelberg, Germany) OCT scanning. Diabetic ME was diagnosed if the OCT foveal thickness (FT) was ≥250 μm on *Stratus-OCT^®^* measurements 23. This latter piece of equipment was used to classify ME, due to the consolidation of its measurements in patients with DR by other published trials.

### Choroidal Thickness Measurement

The *Heidelberg Spectralis* spectral-domain OCT (SD-OCT) at 870 nm with enhanced depth imaging (EDI) was used to measure macular CT, defined as the vertical distance between the posterior edge of the retinal pigment epithelium to the choroidal-scleral junction. CT measurements at 500-μm intervals up to 2,500 μm from the temporal and nasal to the fovea were used for comparisons. An investigator blinded to the study design performed all measurements in one afternoon. On a different occasion, another examiner independently measured the same group of OCTs. To calculate intra- and inter-observer variability, the same two observers repeated their measurements unaware of the previous results.

The fourth-generation OCT employed was equipped with a camera that can be used to monitor the stability of patient fixation. Therefore, the tomograms should occur at the same point. The images were generated using six macular radial scans, centered at the fovea at equally spaced angular orientations, with 20 raster lines spaced 200 μm apart. The machine rated the quality of the images, and only ratings greater than 25 were considered high-quality scans. Cross-sectional images were analyzed using built-in software.

### Study design, treatment and follow-up protocol

**Figure 1:**
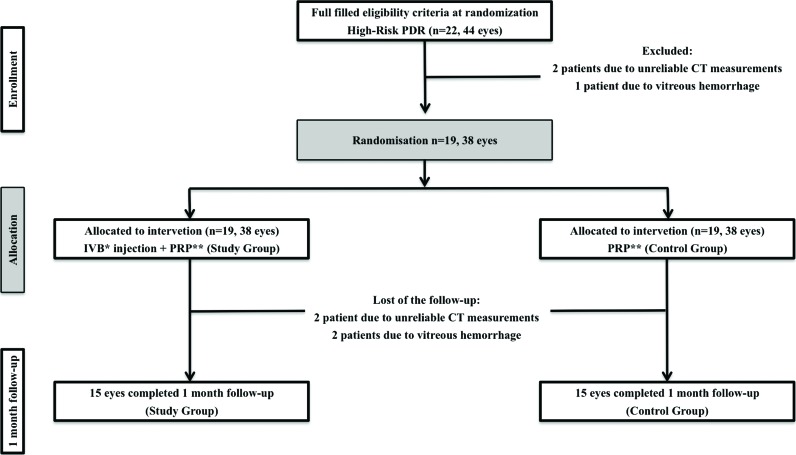
Schematic representation of the study protocol (between-group comparison). *intravitreal bevacizumab; **pan-retinal photocoagulation.

Both eyes of each patient were included. The first eye was randomly assigned to the study group to receive PRP with IVB injections, and the other eye was enrolled in the control group and received PRP alone. A schematic representation of the study protocol is shown in Figure 1.

The control group received PRP once per week for 3 consecutive weeks beginning on the same day as randomization. Between 300 and 500 shots were given per episode. The retinal laser parameters were a spot size of 250 μm, an exposure time between 0.1 and 0.2 msec, and a moderate intensity (200 to 500 mW)^24^. When concomitant DME was present, it was treated during the first episode of PRP based on Olk et al. 25 and the ETDRS guidelines 26.

In the study group, on the day of randomization, one eye received an IVB (1.25 mg/0.05 mL) injection following standard international guidelines 27. The first PRP episode was performed after only 1 week, and the second IVB injection was administered at the end of the third PRP episode. Therefore, the interval between the two injections was 3 weeks, and three episodes of PRP were also performed.

The laser treatment for both eyes was performed after ophthalmic evaluation using a double-frequency Nd:YAG laser (532 nm; Alcon, Ophthalas 532 EyeLite Laser Photocoagulator, Fort Worth, TX, USA) via a 160 Mainster OMRA-PRP lens (Volk Instruments, Bellevue, CA, USA).

The patients were followed for 1 month. Ophthalmic examinations at the end of a 1-month follow-up visit included intraocular pressure measurements using a Goldmann applanation tonometer, slit-lamp biomicroscopy, fundus examination, OCT scanning and fluorescein angiography.

### Study outcomes

The primary outcome was a determination of macular CT measurements of the eyes in the experimental and control groups at baseline and at a 1-month follow-up; these measurements were then compared between the two groups.

The secondary outcome included a longitudinal comparison of macular CT measurements within each group, comparing the measurements at baseline with those at the first-month follow-up visit.

Potential injection-related local side effects, such as ocular hypertension, lens opacity progression, and anterior chamber reaction, were reported to evaluate ocular safety. Arterial thromboembolic events were reported to evaluate systemic safety.

### Statistical analyses

Descriptive statistics included the mean±standard deviation. The Shapiro–Wilk test was used to evaluate the normality assumption. Comparisons of macular CT measurements between and within groups were assessed using the rank-sum and the Wilcoxon signed-rank test, respectively. An intra-class correlation coefficient (ICC) was calculated to assess inter- and intra-rater variability for measurements of macular CT. Fisher’s exact test was applied to compare the number of significantly different measurements in the treatment and the control group. The level of statistical significance was defined as *p*<0.05. The analyses were performed using SPSS, version 15.0 (SPSS Inc., Chicago, IL, USA).

## RESULTS

Twenty-two patients were eligible, and 19 were included in the study. Three patients were excluded before randomization, 2 because of unreliable CT measurements and one because of vitreous hemorrhage. Nineteen patients (38 eyes) were randomized and treated, but at the one-month follow-up, 4 patients were excluded (2 because of vitreous hemorrhage and 2 because of unreliable CT measurements at follow-up). The fourth excluded patient had one eye (from the control group) excluded due to unreliable CT measurements, whereas the eye subjected to PRP and IVB injections was suitable for study.

The intra-class correlation coefficient for the macular CT measurements by the second observer at the fovea, 500 μm nasal and 500 μm temporal was 0.99 for all three measurements (*p*<0.001), indicating excellent inter-observer variability. The corresponding value for repeated measurements by the same reader was 0.98, indicating excellent intra-observer variability (*p*<0.001).

The mean age ± standard deviation of the 19 patients was 53.4±9.3 years, and 9 patients (60%) were males. The average glycated hemoglobin level was 8.89 (range 7.5-9.7). DM had lasted 17.3±8.7 years, and systemic arterial hypertension had lasted 7.3±6.2 years. The mean initial BCVA was 0.21±0.21 and 0.21±0.21 logMAR for the study and control groups, respectively.

DME was present in 11 and 10 eyes of the 15 patients from the study and control groups, respectively. Table 1 shows average FT measurements for both groups throughout the study. A significant increase in FT measurements at a 1-month follow-up, compared to baseline values, was observed in both groups.

### Between-group comparison

Table 2 shows all CT measurements at baseline and at a 1-month follow-up both in the study and in the control group. The comparison between the two groups is based on 19 patients at baseline and 15 patients at a 1-month follow-up (Figure 1). In both groups, CT measurements were greater for the foveal measurement (341.68±67.66 μm and 345.79±83.66 μm for the study and control groups, respectively). The CT increased toward the temporal and decreased toward the nasal regions from the center of the macula. No significant difference was found in any comparison between the two groups at baseline and at the 1-month follow-up measurement.

### Within-group comparison

**Figure 2:**
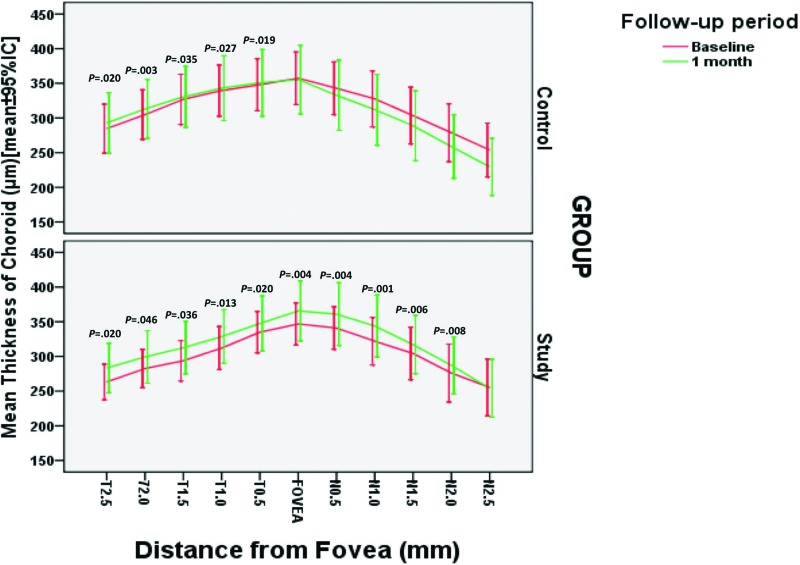
Mean macular choroidal thickness measurements from each group at different locations across a horizontal section through the fovea. This measurement significantly differed at the 1-month follow-up visit compared with the baseline. The error bars represent 1 standard deviation.

Table 3 and Figure 2 show an intra-group comparison of all macular CT measurements before and 1 month after treatment. For intra-group comparisons, 16 eyes in the study group and 15 eyes in the control groups were evaluated before treatment and at the 1-month follow-up. Almost all macular CT measurements taken in the study group increased significantly from the baseline when compared to the 1-month follow-up visit measurement. In contrast, in the control group, only 5 temporal macular CT measurements were significantly higher at the 1-month follow-up. Statistical analysis indicated a significantly greater number of increased measurements in the treatment group than in the control group (*p*=0.024, Fisher’s exact test).

Throughout the study, we did not detect ocular hypertension, lens opacity progression, anterior chamber reactions, or thromboembolic events.

## DISCUSSION

The relationship between the choroid and DR and the effects of PRP with or without intraocular anti-VEGF agents on macular CT are not well understood in high-risk PDR patients. There is conflicting evidence on the effects of anti-VEGF agents on macular CT, even in patients not subjected to PRP. Nevertheless, in PDR diabetic patients, because PRP can increase macular retinal thickness in the short-term, it is possible that it may also alter macular CT. Furthermore, in a prospective, non-comparative, 1-month follow-up study, Takashi et al. 28 observed that PRP significantly increased choroidal blood volume (CBV) and choroidal blood flow (CBF) in the foveal region of patients with DR. The authors suggested that the increased CBV and CBF after PRP may be explained by local inflammation and cytokine (e.g., VEGF) release induced by PRP and/or the regional redistribution of blood flow toward the macula triggered by PRP 28. In addition, the choriocapillaris and large choroidal vessel present VEGF receptors 29; VEGF released soon after PRP can act in choroidal vessels, promoting their dilation and consequently leading to increased thickness.

Zhang et al. 30 reported that PDR patients who underwent PRP had an increased CT soon after the completion of PRP, whereas in DME patients, who likely had higher concentrations of intraocular VEGF, a significant increase was not observed. In contrast to this study, a study by Hwang et al. 31 did not find a significant difference in macular CT after PRP in early-PDR patients, mostly without DME. In the current study, we found that eyes subjected to PRP and two IVB injections had a significantly greater macular CT increase from baseline to the 1-month follow-up than eyes in the PRP-alone group (Table 3 and Figure 2).

Roohipoor et al. 19 reported that eyes with PDR treated with IVB in addition to PRP had a macular CT that decreased 3 months after the procedure, although no significant abnormality was observed at a 1-month post-treatment measurement. However, they did not classify the stage of PDR, and their sample included only eyes without DME. In contrast, in the current study, we found a large majority of patients with DME and high-risk PDR, likely with high concentrations of cytokines, including VEGF. Differences in patient selection and the different effects of the anti-VEGF drug on the retina and the choroid may explain the differences in the study results.

We believe that the finding of a greater CT increase at the end of 1 month in the group subjected to PRP + IVB injections compared to the group subjected to PRP alone may be primarily explained by four mechanisms: a) PRP temporarily increases the macular CBV and CBF 28; b) increased VEGF concentrations in the choroid, increased by the laser treatment, may not be completely neutralized in the choroid by bevacizumab treatment; c) choroidal effusion is generated by choriocapillaris disruption 32; and d) there is a possible stabilization effect of bevacizumab on the external hemato–retinal barrier (EHB) via blockage of VEGF. Elevated VEGF levels induced by laser treatment may cause a breakdown of the EHB by allowing a free liquid shift from and to the choroid and retina; bevacizumab may at least partially restore EHB function. Because it is known that PRP promotes a shift of liquid from the periphery of the choroid to the macular area 8, the bevacizumab-induced stabilization of EHB function may prevent the liquid from moving from the choroid to the retina.

We therefore believe that because laser treatment causes increased VEGF levels inside the eye leading to a more significant breakdown of the EHB, fluid may shift from the choroid to the retina, particularly in eyes with long-standing DME, which have retinal pigment epithelium that has been progressively damaged by the presence of fluid. The use of anti-VEGF bevacizumab treatment as an adjuvant may, in part, restore the hemato–retinal barrier function, somehow preventing a fluid shift and therefore contributing to an increase in choroid thickness, relative to pretreatment measurements in the study group. This reasoning may explain why the control group showed less significant increases in macular CT measurements compared to the treatment group in the current study; this may also explain why Regatieri et al. 18 observed macular CT decreases in patients with long-standing DME and presumably damaged EHB function.

We understand that the limitations of the present study include its relatively small sample size, its short follow-up period and its inclusion of patients with and without DME. However, we believe that the results of this pilot study may stimulate further research on the choroidal contribution in the appearance of DME after PRP.

In conclusion, although significant between-group differences in the macular CT measurements were not observed, our study indicates that when IVB injections were used as an adjuvant to PRP in the treatment of PDR, a more pronounced increase in the macular CT was observed over a brief follow-up. Additional larger studies are required to confirm these findings.

## AUTHOR CONTRIBUTIONS

Preti RC conceived and designed the study, was responsible for data acquisition, analysis and interpretation, and manuscript drafting. Takahashi WY conceived and designed the study, and was responsible for the manuscript critical revision. Mutti A and Ferraz DA were responsible for data acquisition. Nakashima Y was responsible for data analysis and interpretation. Zacharias LC was responsible for data analysis and interpretation, and manuscript drafting. Monteiro ML was responsible for the manuscript drafting and critical revision.

## Figures and Tables

**Table 1:** Foveal thickness measurements for both groups throughout the study.

Groups	Time Comparison	Foveal thickness (µm)	*p*
Study	**Baseline - 1 month (16 eyes)**	**315.56±87.74 - 324.88±68.39**	0.029*
Control	**Baseline - 1 month (15 eyes)**	**288.20±64.25 - 310.27±90.17**	0.011*

* *p*<0.05

**Table 2:** Comparison between macular choroidal thickness measurements of eyes with diabetic retinopathy from the study group and the control group at baseline and at a 1-month follow-up after treatment.

Choroidal thickness measurements (μm)												
Time	Group	N2500	N2000	N1500	N1000	N500	Fovea	T500	T1000	T1500	T2000	T2500
Baseline	**Study**	**252.8**	**274.8**	**300.6**	**318.3**	**336.8**	**341.7**	**327.8**	**304.1**	**287.4**	**279.2**	**264,89**
(n=19)	**Control**	**246.2**	**268.5**	**290.7**	**313.7**	**329.7**	**345.8**	**335.3**	**326.9**	**311.5**	**286.2**	**265.3**
1 month	**Study**	**251.4**	**281.7**	**306.5**	**333.9**	**350.9**	**355.7**	**337.6**	**319.5**	**304.1**	**295.7**	**284.5**
(n=15)	**Control**	**234.9**	**265.2**	**297.1**	**320.1**	**341.6**	**364.5**	**358.7**	**348.8**	**336.8**	**319.9**	**299.4**

* *p*<0.05;

CT = choroidal thickness; N = nasal; and T = temporal

**Table 3:** Macular choroidal thickness of the nasal, temporal and subfoveal regions at baseline and follow-up throughout the study. Comparison within groups.

Choroidal thickness measurements (μm)												
Groups		N2500	N2000	N1500	N1000	N500	Fovea	T500	T1000	T1500	T2000	T2500
Study	**Baseline**	**243.75**	**261.31**	**290.63**	**309.19**	328.19	334.88	326.44	**304.38**	**289.94**	**281.06**	**261.25**
(n=16)	**1 month**	**250.19**	282.88*	313.81*	340.19*	357.19*	362.50*	345.00*	327.31*	312.25*	299.94*	282.81*
Control	**Baseline**	**235.07**	**264.20**	**286.47**	**312.73**	330.33	347.73	338.47	**332.67**	**320.40**	**291.67**	**269.53**
(n=15)	**1 month**	**234.93**	**265.20**	**297.07**	**320.13**	341.60	364.53	358.67*	348.80*	336.80*	319.87*	299.40*

* *p*<0.05;

CT = choroidal thickness; N = nasal and T = temporal
